# Integration or separation? Spatial and temporal representations of whole-body movements in visual working memory

**DOI:** 10.3758/s13421-022-01387-y

**Published:** 2023-01-09

**Authors:** Shiau-Chuen Chiou

**Affiliations:** 1 Neurocognition and Action Research Group, Center for Cognitive Interaction Technology (CITEC), Bielefeld University, Inspiration 1, 33619 Bielefeld, Germany; 2 Faculty of Psychology and Sports Science, Bielefeld University, Bielefeld, Germany

**Keywords:** Working memory, Feature integration, Spatial, Temporal, Whole-body movement

## Abstract

Spatial and temporal information are two major feature dimensions of human movements. How these two types of information are represented in working memory—whether as integrated units or as individual features—influences how much information might be retained and how the retained information might be manipulated. In this study, we investigated how spatial (path/trajectory) and temporal (speed/rhythm) information of complex whole-body movements are represented in working memory under a more ecologically valid condition wherein the spatiotemporal continuity of movement sequences was considered. We found that the spatial and temporal information are not automatically integrated but share the storage capacity and compete for a common pool of cognitive resources. The finding rejects the strong form of object-based representation and supports the partial independence of spatial and temporal processing. Nevertheless, we also found that contextual factors, such as the way movements are organized and displayed, can further modulate the level of object-based representation and spatiotemporal integration.

## Introduction

One of the classic debates in visual working memory research is whether object information is represented as integrated units or as individual features. Specifically, “object-based” representation suggests an automatic integration of features into objects (Luck & Vogel, 1997; Vogel et al., 2001), while “feature-based” representation proposes that features belonging to different feature dimensions are represented separately (Bays et al., 2011; Fougnie et al., 2013; Fougnie & Alvarez, 2011; Wheeler & Treisman, 2002), and thus combining features into objects is resource demanding (Wheeler & Treisman, 2002) and may require a specific storage mechanism (i.e., episodic buffer; Allen et al., 2006; Baddeley, 2000; Hitch et al., 2020). Different from static objects, human movements are dynamic in nature and were shown to have an independent working memory storage from that for objects or for spatial locations (Smyth et al., 1988; Smyth & Pendleton, 1989; Wood, 2007). It is therefore intriguing to ask whether different features of a movement are retained as an integrated representation or as individual features in working memory.

In one pioneer study, Wood (2007) used computer-animated human actions as visual stimuli and compared working memory for individual action properties (e.g., action type, duration, side of the body) with working memory for actions defined by multiple properties. Based on a change-detection method adapted from Luck and Vogel (1997), it was assumed that if actions are retained as integrated representations, performance should remain similar regardless of the number of properties being retained for each action. On the contrary, if actions are stored as individual properties, performance should decline when more properties are required to be retained for each action. The results showed that participants can memorize nine properties distributed across three actions as well as three properties distributed across three actions. The finding therefore suggests that features of movements are stored as integrated representations in working memory, consistent with the view of the object-based representation (Luck & Vogel, 1997; Vogel et al., 2001).

Different from action properties, information about identities of agents (Wood, 2008), scenes (Urgolites & Wood, 2013), or visual features (e.g., color) of actions (Ding et al., 2015) were shown to be retained separately from actions, and thus integrating agent and action, scene and action, or color and action is resource demanding. Neurophysiological studies also provided evidence in support of this finding by showing distinct cortical pathways for processing object information, spatial information, visually guided actions, and navigationally relevant information (e.g., scene) (see Kravitz et al., 2011, for a review). In general, previous research suggests that visual working memory stores features that are inherent to actions as integrated representations, but stores actions and other nonaction features (e.g., agent identity, color) or information (e.g., scene) separately.

However, by presenting isolated, independent actions one after another in the change-detection task, most of the previous research implicitly assumed that human actions are “object-like” in nature, without considering potential influence from spatiotemporal dependence between movement units (i.e., spatiotemporal continuity of movement sequences)—one of the most important characteristics of human movements. It was also neglected that movement features (e.g., path, speed), given the dynamic nature, can also be integrated over time. Specifically, spatiotemporal continuity may encourage integration *within* respective feature dimensions across temporally adjacent movement units (“feature-based” integration), while discouraging integration *across* feature dimensions within individual movement units (“object-based” integration). Therefore, presenting actions as isolated units may increase the extent to which features are bound into integrated representations, as illustrated by Wood (2008) that the separately stored agent and action information were bound into more integrated units with the presence of visual cues that differentiated the agents. In other words, the object-based representation previously observed on movement features (i.e., intrinsic properties of movements) might be a special case in which integration is strengthened by the discrete/discontinuous display of movements.

To test this hypothesis, we investigated how spatial (path/trajectory) and temporal (speed/rhythm) information of movements are retained in working memory—whether they are retained as integrated representations or as individual features—under a more ecologically valid condition wherein the spatiotemporal continuity of movement sequences is considered. Specifically, instead of presenting isolated, independent movement units one after another, we linked the ending pose of the first unit with the starting pose of the second one, and so on, to create a relatively continuous movement sequence. Since movement units were all without interpretable external goals and action semantics, we did not presume any higher-order representation to be formed due to the spatiotemporal continuity except for the possibility of perceptual integration between the adjacent movement units. Note that the spatial and temporal information we investigated in the present research are intrinsic properties of movements, namely, movement trajectory and rhythm in specific, rather than space and time in general. Therefore, we did not tackle questions, for instance, whether the information about *where* (space) and *when* (time) a movement occurred might be integrated with the movement itself in memory.

In Experiment 1, participants performed a change-detection task (same/different judgment) on whole-body movement sequences under three conditions: (1) *Temporal-only*, in which participants were informed that only temporal changes could occur and thus spatial information was task-irrelevant. (2) *Spatial-only*, in which participants were informed that only spatial changes could occur and thus temporal information was task-irrelevant. (3) *Both*, in which participants were informed that a change could occur in *either* spatial or temporal domain and thus both spatial and temporal information were task-relevant. We compared participants’ performance when only one feature dimension was task-relevant (i.e., single-processing condition, including the *Temporal-only* and *Spatial-only* conditions) with that when both feature dimensions were task-relevant (i.e., dual-processing condition, including temporal and spatial trials in the *Both* condition).

If spatial and temporal information are automatically integrated without additional cost, performance should remain similar between the single-processing and dual-processing conditions in both spatial and temporal domains. Note that, however, a lack of performance decline would not necessarily imply that the spatial and temporal information are bound as an integrated unit, as it might alternatively indicate that the two features are stored in parallel systems each with separate capacities (Wheeler & Treisman, 2002). On the contrary, if spatial and temporal information are retained as individual features, performance should decline in the dual-processing condition if the two features compete for the same storage capacity, or, as indicated previously, performance might remain the same if the two features are stored in parallel systems. To differentiate between the latter two possibilities, we further manipulated the sequence length (i.e., the number of movement units in a sequence) as being two units or four units. Since working memory capacity for human movements were shown to be two to four units (Shen et al., 2014; Wood, 2007), retaining two units of spatial and temporal information each should not have exceeded respective capacities in parallel systems (if any). However, if spatial and temporal information share the same storage capacity or processing resources, retaining two units of spatial and temporal information each (i.e., four units in total) might have surpassed the common capacity. We hypothesized that the spatiotemporal continuity would encourage feature-specific integration across adjacent movement units and discourage unit-based integration across feature dimensions, leading to a performance decline in the dual-processing condition especially when the sequence length was long (i.e., four units).

## Experiment 1

### Method

#### Participants

Thirty-two participants were recruited for the experiment. One was excluded from analyses due to below-chance performance (out of 2 standard deviations from the group mean), leaving a final sample of 31 participants (16 female; ages 18–34 years, *M* = 25.4, *SD* = 4.3). The original sample size of 32 was determined based on a power analysis (using G*Power 3.1; Faul et al., 2007) to provide a power of .90 at an alpha level of .05 to detect a moderate- to large-sized effect of dual processing (*d* = 0.6) for within-subjects comparisons. Participants’ experiences in dance, music, and sport were evaluated by a questionnaire and reported here as expertise indexes (0: No experience, 1: Beginner, 2: Intermediate amateur, 3: Advanced amateur, 4: Professional) of 0.4 (*SD* = 0.6), 0.7 (*SD* = 0.8), and 1.3 (*SD* = 1.0), respectively, defined by both the training length and skill level.^1^ No professionals were recruited in the present research. Each participant signed informed consent prior to the experiment and received €8 per hour or in exchange for course credit for their participation. The two experiments presented in this paper were conducted in accordance with the ethical principles stated within the declaration of Helsinki (1964) and were approved by the Ethics Committee of Bielefeld University.

#### Stimuli and apparatus

Twenty-four whole-body movement sequences (half performed by a female dancer and the other half performed by a male dancer, both in fitted black clothing), selected from the stimulus set of Chiou (2022a), were used in the current study. No participants had viewed the movement sequences before.

**Fig. 1:**
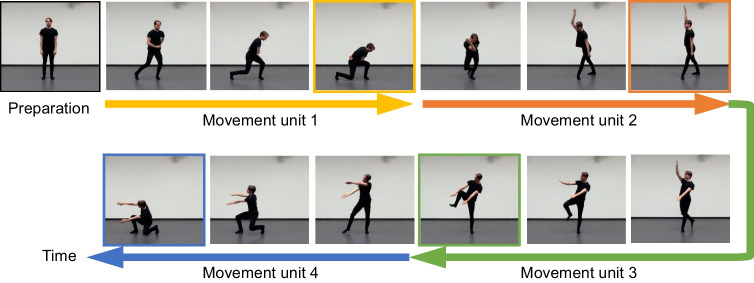
1 Illustration of a whole-body movement sequence. Reprinted from Chiou (2022a) (CC BY 4.0). Three sample video clips (A_2132, A_3212, B_3212) that illustrate movement sequences with different trajectories (A, B) and/or rhythms (2132, 3212) were published with the article Chiou (2022a) and can be seen online:  10.1007/s10339-022-01078-1

Each sequence was composed of four linked movement units, all without interpretable external goals and action semantics. A movement unit was defined as a coordinated whole-body movement that can be performed with a bell-shaped velocity profile (i.e., accelerating till the midpoint of the movement and then decelerating; Abend et al., 1982)^2^ and thus had a clear starting point and ending point where the velocity was zero (see Fig. 1 for an illustration). In addition, each sequence was performed in four metric complex rhythms (i.e., integer-ratio rhythms without regular temporal accents aligned with the beat): 3212, 2132, 1223, and 2321. Metric complex rhythms were shown to be more difficult to induce beat perception (Grahn, 2012) and thus were used here to avoid beat-based encoding. Each rhythm was composed of four temporal durations (one 1-beat duration, two 2-beat durations, and one 3-beat duration), corresponding to four movement units of a sequence. When the same movement unit was performed, a shorter duration also implied a higher speed. The rhythms were paced at a tempo of 90 beats per minute, yielding a sequence length of around 6 s after including one additional beat for preparation purpose at the beginning of the sequence.

Movement recordings were made with a digital video camera recorder (Sony HDR-CX430V) at 50 frames per second against a white background and a gray floor. Videos were then edited on a frame basis using the software iMovie (Apple, Inc.) and presented silently to participants at 1,600 × 900 pixels on a 24-inch LCD screen (Dell U2412M) with a viewing distance of approximately 50 cm. The experimental flow and data processing were programmed in Python; stimuli presentation was implemented with the PsychoPy software package (Peirce, 2007, 2009).

#### Design and procedure

**Fig. 2:**
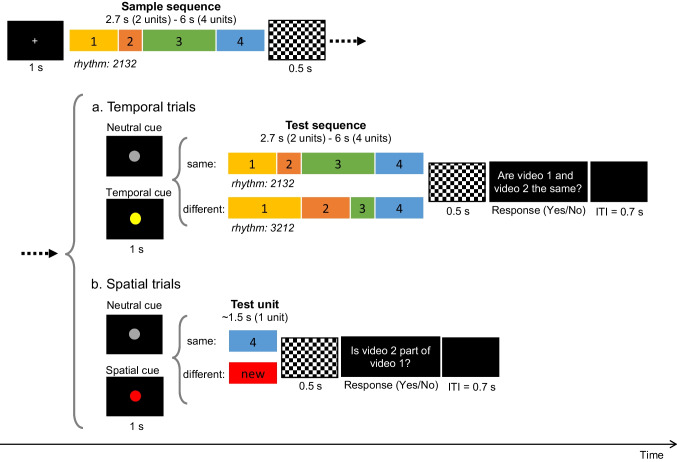
Trial structures of Experiment 1 with spatiotemporal-continuous movement sequences (1234) as visual stimuli. The whole-display paradigm was used for temporal trials (**a**), in which the sample and the test sequences were of the same length (i.e., 2 or 4 units). The single-probe paradigm was used for spatial trials (**b**), in which participants judged whether the one-unit test probe was part of the sample sequence. The sample sequence was followed by a mask for 0.5 s and a 100%-valid response cue. A neutral cue (a gray circle) was used in the single-processing condition (i.e., *Temporal-only* and *Spatial-only* conditions), while a temporal cue (a yellow circle) or a spatial cue (a red circle) was used in the dual-processing condition (i.e., *Both* condition) to indicate in which dimension a potential change might occur and thus whether a test sequence (in temporal trials) or a test unit (in spatial trials) would be displayed thereafter. ITI indicates intertrial interval. (Color figure online)

Participants performed a change-detection task (same/different judgment) with a procedure similar to Chiou (2022a) under three conditions: (1) *Temporal-only*, in which participants were instructed to attend to the temporal information (“movement speed”) of the sample sequence and detect whether a temporal change occurred in the test sequence (Fig. 2a). The whole-display paradigm was used in temporal trials (i.e., trials with potential changes in the temporal domain), as temporal durations were difficult to discriminate individually if without beat perception. The spatial information (“movement path”) was kept unchanged in temporal trials. (2) *Spatial-only*, in which participants were instructed to attend to the spatial information of the sample sequence and judge whether the test unit was part of the sample sequence (Fig. 2b). The single-probe paradigm was used in spatial trials (i.e., trials with potential changes in the spatial domain) to ensure that participants encoded each unit of a sequence. Note that the whole-display paradigm was not applicable under this condition, since a change in any unit of the sequence would make the continuous trajectory into a discontinuous one and thus be easily detectable even without memory involvement. The temporal duration was kept unchanged for the test probe that belonged to the sample sequence. (3) *Both*, in which participants were instructed to attend to both the temporal and spatial information and detect a potential change in *either* domain. The words *path* and *speed* (instead of *trajectory* and *rhythm*) were used in verbal instructions to make the concepts more understandable to participants. The three experimental conditions were performed in three separate sessions with an order first counterbalanced between the single-processing (i.e., *Temporal-only*, *Spatial-only*) and dual-processing (i.e., *Both*) conditions and then between the two single-processing conditions across participants.

Each trial began with a 1-s fixation cross (+), followed by a sample sequence of two or four units. To control for the starting pose, a two-unit sequence was defined as the first-half of a four-unit sequence (i.e., Unit 1 + Unit 2). A mask with a black–white chessboard pattern was presented for 0.5 s after the offset of the sample sequence, followed by a 100%-valid response cue and a test sequence (or a test unit). A neutral cue (a gray circle with 0.6 degree in diameter) was used in the single-processing condition, while a temporal cue (a yellow circle with 0.6 degree in diameter) or a spatial cue (a red circle with 0.6 degree in diameter) was used in the dual-processing condition to indicate in which feature dimension a potential change might occur and thus whether a test sequence (in temporal trials) or a test unit (in spatial trials) would be displayed thereafter. The partial-report method was adopted to reduce the decision noise when comparing multiple features in the change-detection task (Shin & Ma, 2017) and to avoid confusion due to the inconsistent display between temporal and spatial trials. After the offset of the test sequence (or the test unit), a mask for 0.5 s was presented, followed by a question *“Are video 1 and video 2 the same?”* (in temporal trials) or “*Is video 2 part of video 1?*” (in spatial trials) (see Fig. 2a–b)*.* Participants were required to make a yes/no judgment by keystroke on a standard computer keyboard (“F” key and “J” key, respectively, marked in red). They were instructed to respond as accurately as possible without a strict time constraint. For two-unit sequences, the temporal contrast of 23 (two beats plus three beats) versus 32 (three beats plus two beats) was not included in the experimental trials due to less perceptual salience (i.e., more difficult to discriminate).

Participants completed eight practice trials before the start of each session. In practice trials, participants were allowed to replay the sample sequence before making a judgment and received feedback (as shown by the word *correct* or *incorrect* on the computer screen) on a trial-by-trial basis. No video replay or feedback was provided in formal experiment. Overall, participants performed 48 trials in the *Temporal-only* condition, 48 trials in the *Spatial-only* condition, and 96 trials in the *Both* condition (all with 50% *different* trials). Twenty-four movement sequences were shuffled on an individual subject basis and equally divided into the *Temporal-only* and *Spatial-only* conditions. The sequences then served reversely in the *Both* condition; namely, sequences used in the *Temporal-only* condition would be the spatial trials in the *Both* condition, and sequences used in the *Spatial-only* condition would be the temporal trials in the *Both* condition. The goal of this arrangement was to diminish potential influence from the attention-based familiarity. The entire experiment lasted about 60 minutes, including short breaks within and between sessions.

### Results

We calculated proportion of correct responses for temporal and spatial trials, respectively, as a key performance measure, defined as a mean of the hit rate (correctly responding “same” on *same* trials) and correct-rejection rate (correctly responding “different” on *different* trials). The statistical threshold of Type I error was set at α = .05, Cohen’s *d* and partial eta squared (η_p_^2^) were reported to indicate effect size, and post hoc analyses were conducted using Bonferroni correction. Statistical analyses were performed with the software JASP (Version 0.11.1; JASP Team, 2019).

#### Spatial and temporal information of movements were not automatically integrated

**Fig. 3:**
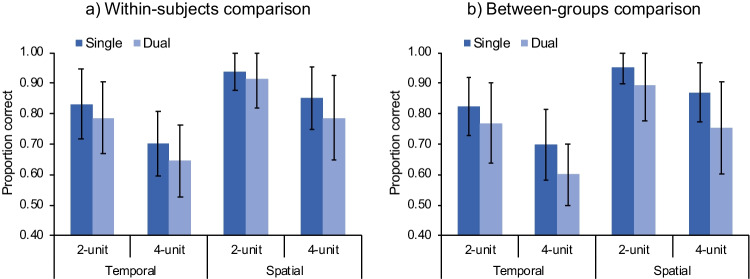
Change-detection performance (measured by proportion of correct responses) in Experiment 1 for within-subjects comparison (**a**) and between-groups comparison (**b**) of processing type (single, dual). Error bars indicate one standard deviation of the mean

Data were first analyzed by a 2 (processing type: single, dual) × 2 (information type: temporal, spatial) × 2 (sequence length: two units, four units) × 2 (order: single + dual, dual + single) mixed analysis of variance (ANOVA) on proportion correct, with “order” as a between-subjects factor. The factor “order” was added to check whether there was any effect due to the order of performing the single-processing and dual-processing conditions. The results showed significant main effects for processing type, *F*(1, 29) = 24.2, *p* < .001, η_p_^2^ = .46, information type, *F*(1, 29) = 176, *p* < .001, η_p_^2^ = .86, and sequence length, *F*(1, 29) = 145, *p* < .001, η_p_^2^ = .83. Participants performed better in the single-processing (*M* = 83.1%, 95% CI [80.3%, 85.9%]) than in dual-processing (*M* = 78.2%, 95% CI [75.4%, 81.0%]) conditions, *t*(30) = −4.54, *p* < .001, *d* = −0.82, when the sequence length was two units (*M* = 86.7%, 95% CI [84.0%, 89.5%]) than four units (*M* = 74.6%, 95% CI [71.8%, 77.4%]), *t*(30) = −11.8, *p* < .001, *d* = −2.13, and for spatial (*M* = 87.2%, 95% CI [84.4%, 90.0%]) than for temporal (*M* = 74.1%, 95% CI [71.4%, 76.9%]) information, *t*(30) = 13.3, *p* < .001, *d* = 2.40 (Fig. 3a). A significant performance decline from the single- to dual-processing conditions (i.e., dual-processing cost) indicates that the spatial and temporal information were not automatically integrated.

It is worth noting that the interaction effect between processing type and order was also significant, *F*(1, 29) = 5.26, *p* = .029, η_p_^2^ = .15. The dual-processing cost was smaller (i.e., nonsignificant) in the order group “single + dual,” mean difference = −2.6%, *p*_*bonf*_ = .415, *d* = −0.34, in comparison with that in the “dual + single” group, mean difference = −7.2%, *p*_*bonf*_ < .001, *d* = −0.90. The finding illustrates potential learning effects when participants started with the single-processing condition, followed by the dual-processing condition, but not vice versa. Specifically, participants in the “single + dual” group might have learned how to process two information streams simultaneously from their experience of processing each information stream separately. No other effects were significant, all *p*s ≥ .142.

One may suspect that the learning effect was due to insufficient practice before the formal experiment. However, based on our previous experience (e.g., Chiou, 2022a, 2022b), eight practice trials (with replay and feedback) should be sufficient for participants to understand and to perform the task properly. Moreover, in one of our previous studies (Chiou & Schack, 2023), participants performed a similar change-detection task under a dual-processing condition for 288 trials (much more than the number of trials performed in the present study), but no learning effect was found across two experiments (*N* = 54 in total). The finding suggests that participants’ sensitivity to spatial/temporal changes of the current stimulus set was relatively stable, and therefore the learning effect observed here should be attributed more to the development of a processing strategy than the increase of familiarity to the task.

**Fig. 4:**
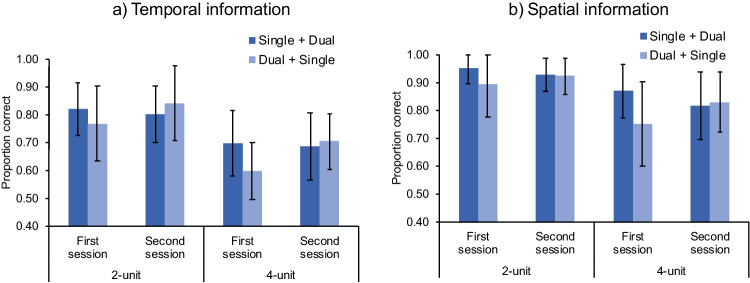
Change-detection performance (measured by proportion of correct responses) of the order groups “single + dual” and “dual + single” in temporal (**a**) and spatial (**b**) domains in Experiment 1. First-session performance of the “single + dual” group was the performance under the single-processing condition; first-session performance of the “dual + single” group was the performance under the dual-processing condition. The same holds for second-session performance. Error bars indicate one standard deviation of the mean

In addition, as discussed previously, the learning effect was asymmetric. Specifically, the dual-processing condition was performed better, albeit nonsignificant, in the second session by the “single + dual” group (*M* = 80.9%, *SD* = 8.2%) than in the first session by the “dual + single” group (*M* = 75.4%, *SD* = 9.2%), *t*(29) = 1.76, *p* = .090, *d* = 0.63, while the performance under the single-processing condition did not show this tendency (*M* = 82.6%, *SD* = 6.9% in the “dual + single” group; *M* = 83.5%, *SD* = 5.9% in the “single + dual” group), *t*(29) = 0.42, *p* = .679, *d* = 0.15 (Fig. 4). If the current results were indeed due to the unfamiliarity to the task (i.e., insufficient practice), the single-processing performance should also have been boosted (due to the increase of familiarity) when performed by the “dual + single” group in the second session. Yet the data did not support this hypothesis.

To examine the dual-processing cost without potential influence from order (e.g., learning effect), we further conducted a between-groups analysis by comparing the first-session performance of the two order groups, namely the single-processing condition performed by the “single + dual” group and the dual-processing condition performed by the “dual + single” group. A 2 (processing type: single, dual) × 2 (information type: temporal, spatial) × 2 (sequence length: two units, four units) mixed ANOVA on proportion correct, with “processing type” as a between-subjects factor, yielded similar results as did the within-subjects analysis. The main effects were significant for processing type, *F*(1, 29) = 8.65, *p* = .006, η_p_^2^ = .23, information type, *F*(1, 29) = 64.9, *p* < .001, η_p_^2^ = .69, and sequence length, *F*(1, 29) = 81.7, *p* < .001, η_p_^2^ = .74. Participants performed better in the single-processing (*M* = 83.7%, 95% CI [79.7%, 87.6%]) than in dual-processing (*M* = 75.5%, 95% CI [71.6%, 79.5%]) conditions, *t*(29) = 2.94, *p* = .006, *d* = 0.53, indicating that the spatial and temporal information were not encoded automatically as integrated representations. No other effects were significant, all *p*s ≥ .076 (Fig. 3b).

Moreover, due to a performance difference between spatial and temporal processing, we also analyzed the data by taking the base performance into account. We calculated dual-processing costs in the spatial and temporal domains for each participant by using the formula: – [100 × (dual performance – single performance) / single performance]. Positive values indicate a cost under the dual-processing condition, and negative values indicate a benefit. We then conducted a 2 (information type: temporal, spatial) × 2 (sequence length: two units, four units) repeated-measures ANOVA on dual-processing costs. There were no significant effects for information type, *F*(1, 30) = 0.003, *p* = .960, η_p_^2^ = .000, sequence length, *F*(1, 30) = 1.00, *p* = .326, η_p_^2^ = .03, or the two-way interaction, *F*(1, 30) = 0.22, *p* = .646, η_p_^2^ = .01, indicating comparable dual-processing costs between spatial and temporal domains and between sequence lengths of two units and four units.

#### Relative performance influenced dual-processing costs

In addition to the group-level analysis, we also examined if relative advantage of processing spatial or temporal information might influence the prioritization of the two information streams under the dual-processing condition on individual subject level. We computed Pearson product-moment correlation coefficients between relative performance (measured by performance in *Temporal-only* condition minus performance in *Spatial-only* condition; T-S) and dual-processing costs (computed in the same way as above, with positive values indicating a cost) in spatial and temporal domains, respectively. Negative values of T-S indicate a disadvantage of processing temporal information compared to spatial information, while positive values indicate an advantage. The value of T-S was negative for most participants due to a higher sensitivity to spatial than to temporal information of movements based on the current design.

**Fig. 5:**
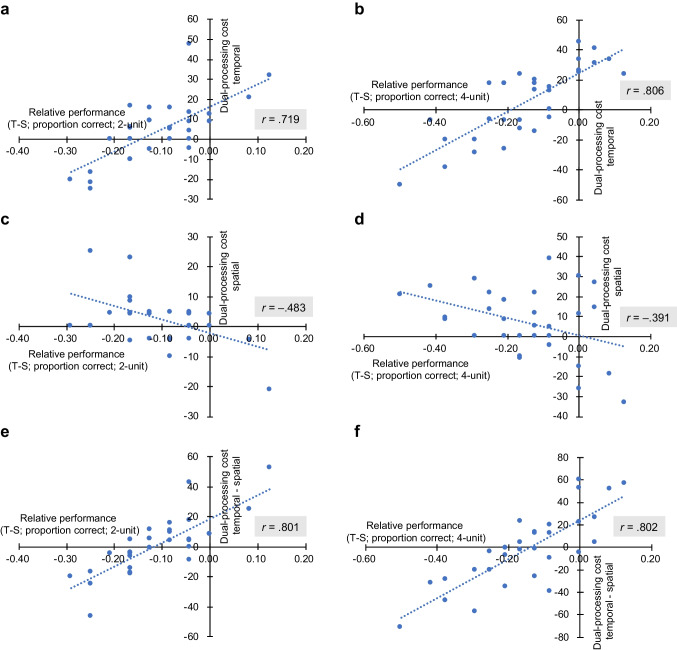
Correlations between relative performance (measured by proportion of correct responses in *Temporal-only* condition minus that in *Spatial-only* condition; T-S) and dual-processing costs in temporal domain (**a–b**), spatial domain (**c–d**), and the difference between the two (**e–f**), when the sequence length was 2-unit and 4-unit. Blue dots represent individual-participant data (*N* = 31). Dual-processing cost was calculated with the formula: – [100 × (dual performance – single performance) / single performance]. Positive values indicate a cost under the dual-processing condition, and negative values indicate a benefit. (Color figure online)

The results showed positive correlations between relative performance (T-S) and dual-processing cost in the temporal domain, *r* = .719, *p* < .001 for two-unit sequences; *r* = .806, *p* < .001 for four-unit sequences (Fig. 5a–b), but negative correlations in the spatial domain, *r* = –.483, *p* = .006 for two-unit sequences; *r* = –.391, *p* = .029 for four-unit sequences (Fig. 5c–d). An increase of disadvantage in temporal compared to spatial processing (i.e., a more negative value of T-S) was thus associated with a decrease of dual-processing cost in the temporal domain but an increase of the cost in the spatial domain. The finding suggests that participants prioritized the processing of information in the worse-performance domain under the dual-processing condition by scarifying the processing of information in the better-performance domain. In other words, participants allocated more cognitive resources to temporal processing when it was relatively worse performed, leading to a higher dual-processing cost in the spatial domain—or, contrarily, a higher dual-processing cost in the temporal domain when temporal information had an advantage in processing (see Fig. 5e–f).

#### Temporal salience did not modulate spatial encoding

Among the three temporal durations (one beat, two beats, three beats) used in the current study, movement units performed with the shortest duration also had the highest speed and thus were the most perceptually salient. In the following analysis, we examined whether the perceptual salience of temporal information might influence memory encoding of the corresponding spatial information. Specifically, if spatial and temporal information were encoded as an integrated representation, temporal salience might modulate spatial encoding; on the contrary, if spatial and temporal information were encoded separately, there would not be cross-domain modulation. We conducted a 3 (beat: one beat, two beats, three beats) × 2 (processing type: single, dual) repeated-measures ANOVA on accuracy of the *same* trials (i.e., hit rate) for spatial judgments. *Different* trials were excluded, as the test probe did not belong to the sample sequence and thus no temporal modulation during encoding could be observed. In addition, to ensure a sufficient number of trials for analysis, factors “order” and “sequence length” were collapsed. The results yielded no significant main effect for beat, *F*(2, 60) = 0.64, *p* = .529, η_p_^2^ = .02, or the interaction effect between beat and processing type, *F*(2, 60) = 0.39, *p* = .682, η_p_^2^ = .01, indicating that the temporal salience did not modulate spatial encoding either in the single-processing condition, in which the corresponding temporal information was task-irrelevant, or in the dual-processing condition, in which the temporal information was consciously attended to. The results therefore suggest that there might be no spatiotemporal integration during spatial processing.

### Discussion

A mutual interference occurred in the dual-processing condition indicates that spatial and temporal information of movements are not *automatically* integrated (i.e., integrated without additional costs in terms of the storage capacity or processing resources). More importantly, there was no modulation from the sequence length to the dual-processing cost in either spatial or temporal domain. Performance deteriorated in the *Both* condition in comparison with that in the *Temporal-only* and *Spatial-only* conditions even when only two units of each information were required to be retained. As working memory capacity for human movements was shown to be two to four units (Shen et al., 2014; Wood, 2007), retaining two units of spatial and temporal information each would not have exceeded respective capacities if there were parallel systems. The finding therefore suggests that spatial and temporal information of movements, albeit not integrated, are not processed fully in parallel.

Our finding is inconsistent with the view of the object-based representation, which proposes that features of an object or an action are automatically integrated and retained as a bound representation in working memory (e.g., Luck & Vogel, 1997; Vogel et al., 2001; Wood, 2007, 2011). However, our finding does not support the pure feature-based representation, either, according to which different features are supposed to have their own independent storage capacities (e.g., Magnussen & Greenlee, 1999; Wheeler & Treisman, 2002). One possible explanation is that, the extent of the dual-processing cost is not only determined by which feature dimension a feature belongs to, but also the level of dependence between individual feature dimensions. It has been suggested that the degree of feature dependence (or independence) is determined by the degree of overlap in neural populations coding the features (Fougnie & Alvarez, 2011). Although spatial and temporal information can be coded by largely independent neurons, the perception of movements entails an integration of spatial information over time (Giese & Poggio, 2003; Lange et al., 2006), suggesting an intrinsic spatiotemporal dependence in the early stage of movement perception. Moreover, as temporal information (speed/rhythm) was defined here as the change of spatial information (path/trajectory) over time (i.e., second-order feature), the subordinate position of the temporal information would also imply an intrinsic dependence of temporal processing on spatial information. As a result, it is not surprising that spatial and temporal information might be partially dependent and share, to some extent, the storage capacity or processing resources. The finding of the “partial independence” of memory capacities for different feature dimensions is also consistent with the consensus view (see Schneegans & Bays, 2019, for a review).

The results of Experiment 1 reject the “strong form” of object-based representation when spatiotemporal-continuous movement sequences were used as visual stimuli. Although the finding might be attributed to the continuous nature of movement sequences that encourages feature-based more than object-based integration, the current results cannot rule out the possibility that the inconsistency with the previous finding supporting spatiotemporal integration (e.g., Wood, 2007) may arise from the higher complexity of movements used in the current study (i.e., coordinated whole-body movements) in comparison with that of those used in previous research (e.g., movements of a single body part, such as arm raise, head turn, leg raise in Wood, 2007). Specifically, complex movements may impose additional demand on perceptual and cognitive systems, changing the way the observed information might be processed. Moreover, complex movements may provide more cues or contextual information that are beneficial for the construction of higher-order representations, increasing the possibilities of how the observed information might be encoded.

To clarify this point, we examined in Experiment 2 whether the spatiotemporal continuity of movement sequences might be the key contributor to the feature-based, albeit partially dependent, processing of spatial and temporal information, or the feature-based representation might be a general phenomenon that can be observed on complex movements. We used the same movement sequences as in Experiment 1, but displayed them in a discontinuous manner. If the feature-based representation is mainly due to the spatiotemporal continuity of movement sequences, the dual-processing cost should be reduced on discontinuous sequences, as the spatiotemporal discontinuity would encourage object-based encoding and spatiotemporal integration. On the contrary, if the feature-based representation is a general phenomenon, the dual-processing cost should remain observable on discontinuous sequences. The same experimental design was used in Experiment 2 as in Experiment 1 (except for a change in sequence display; see the Method section, below). However, to eliminate potential influence from order (i.e., the asymmetric learning effect), each participant only performed one session in Experiment 2, either the single-processing condition (i.e., *Temporal-only* and *Spatial-only*) or the dual-processing condition (i.e., *Both*), and thus only the between-groups analysis was conducted.

## Experiment 2

### Method

#### Participants

Thirty-two new participants were recruited for the experiment and randomly assigned to the single-processing group (“single group”) (*n* = 16, including nine females; ages 20–37 years, *M* = 26.4, *SD* = 4.7) or dual-processing group (“dual group”) (*n* = 16, including 12 females; ages 19–31 years, *M* = 23.9, *SD* = 3.9). Given the same experimental design in Experiments 1 and 2 and a significant between-group effect on processing type that has already been observed in Experiment 1, a sample size of 32, which was the same as the original sample size of Experiment 1, was deemed sufficient to detect a difference, if any, between the single- and dual-processing groups. Participants’ expertise indexes in dance, music, and sport were 0.3 (*SD* = 0.4), 0.6 (*SD* = 0.5), and 1.3 (*SD* = 1.1) in the single group and 0.4 (*SD* = 0.5), 0.6 (*SD* = 0.7), and 1.5 (*SD* = 0.7) in the dual group, indicating limited experiences in the respective fields. There was no between-groups difference in expertise indexes (all *p*s ≥ .462). All participants signed informed consent prior to the experiment and received €8 per hour or in exchange for course credit for their participation.

#### Stimuli, design, and procedure

**Fig. 6:**
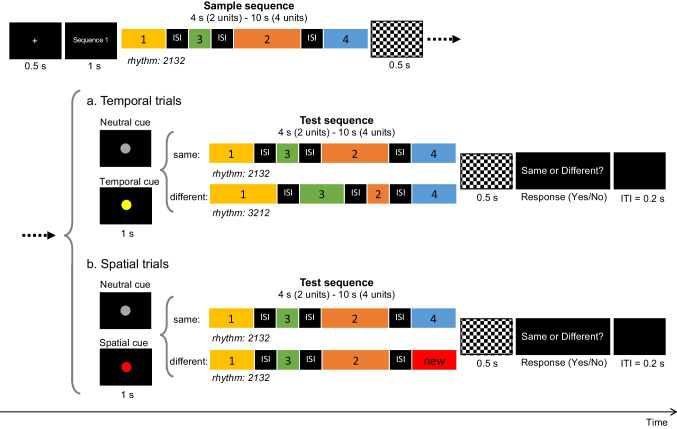
Trial structures of Experiment 2 with spatiotemporal-discontinuous movement sequences (1324) as visual stimuli. The whole-display paradigm was used for both temporal (**a**) and spatial (**b**) trials, in which the sample and the test sequences were of the same length (i.e., 2 or 4 units). The sample sequence was followed by a mask for 0.5 s and a 100%-valid response cue. A neutral cue (a gray circle) was used in the single-processing condition (i.e., *Temporal-only* and *Spatial-only* conditions), while a temporal cue (a yellow circle) or a spatial cue (a red circle) was used in the dual-processing condition (i.e., *Both* condition) to indicate in which dimension a potential change might occur. Interstimulus intervals (ISIs) of 1.25 s were inserted between the movement units of the sequences. ITI indicates intertrial interval. (Color figure online)

The same twenty-four movement sequences were used as in Experiment 1. The display order of the second and the third units of the sequences was switched to create discontinuous trajectories (i.e., spatial discontinuity), and interstimulus intervals (ISIs) of 1.25 s, shown as black screen, were inserted between the movement units to create discontinuity in temporal display (i.e., temporal discontinuity) (see Fig. 6a–b). An ISI of 1.25 s was determined based on one of our previous studies (Chiou, 2022b) showing that an ISI of 1.25 s could balance the working memory demand for movements extracted from a continuous sequence with that for those extracted from a discontinuous sequence when a single-probe change-detection paradigm was used.

Same as Experiment 1, participants in the single group performed the change-detection task under the *Temporal-only* and *Spatial-only* conditions in two separate sessions (with the order counterbalanced between participants), while participants in the dual group performed the task under the *Both* condition in one single session (see the Design and Procedure section of Experiment 1). Different from Experiment 1, the whole-display (rather than single-probe) paradigm was used for spatial trials, in which participants were required to judge whether there was a spatial change in one of the units of the test sequence. The whole display was used for both spatial and temporal trials to reduce the processing cost that may be incurred due to the inconsistent display from trial to trial in the *Both* condition. To avoid confusion, a short text “*Sequence 1*” was added after the fixation cross to indicate that the sample sequence was about to display. The presentation of the question was also simplified to “*Same or Different?*” for both spatial and temporal trials (see Fig. 6a–b).

To equate the number of practice trials between the groups, participants in the single group performed eight practice trials before the start of the *Temporal-only* and *Spatial-only* conditions, while participants in the dual group performed 16 practice trials before the start of the *Both* condition. Other procedures remained the same as in Experiment 1. The entire experiment lasted about 45 minutes, including short breaks within and between sessions.

### Results

**Fig. 7:**
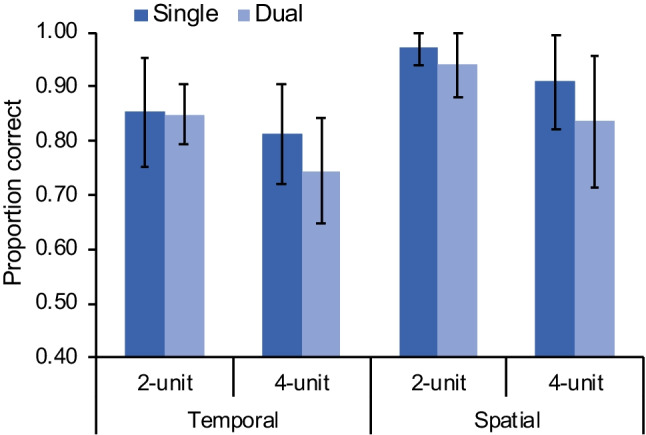
Change-detection performance (measured by proportion of correct responses) in Experiment 2 with processing type (single, dual) as a between-subjects factor. Error bars indicate one standard deviation of the mean

We conducted a 2 (processing type: single, dual) × 2 (information type: temporal, spatial) × 2 (sequence length: two units, four units) mixed ANOVA on proportion correct, with “processing type” as a between-subjects factor. The results showing significant main effects for processing type, *F*(1, 30) = 4.56, *p* = .041, η_p_^2^ = .13, information type, *F*(1, 30) = 39.6, *p* < .001, η_p_^2^ = .57, and sequence length, *F*(1, 30) = 40.0, *p* < .001, η_p_^2^ = .57, were consistent with Experiment 1. However, the interaction effect between processing type and sequence length was also significant, *F*(1, 30) = 4.60, *p* = .040, η_p_^2^ = .13. Post hoc analyses yielded no significant difference between the single and dual groups when the sequence length was two units, mean difference = 1.7%, *p*_*bonf*_ = 1.000, *d* = 0.13, but the difference was significant when the sequence length was four units, mean difference = 7.0%, *p*_*bonf*_ = .030, *d* = 0.52. The results suggest that there was a tendency of object-based representation and within-unit spatiotemporal integration on discontinuous sequences, especially when the sequence length was short (i.e., two units). No other effects were significant, all *p*s ≥ .555 (Fig. 7).

In addition, we conducted a 3 (beat: one beat, two beats, three beats) × 2 (processing type: single, dual) mixed ANOVA on hit rates of the spatial trials, with “processing type” as a between-subjects factor to examine whether temporal salience might modulate spatial encoding. The results yielded no significant main effect for beat, *F*(2, 60) = 0.41, *p* = .667, η_p_^2^ = .01, or the interaction effect between beat and processing type, *F*(2, 60) = 0.95, *p* = .393, η_p_^2^ = .03, indicating no cross-domain modulation from temporal salience on spatial encoding and that spatial processing might be relatively independent from temporal information.

### Discussion

We found a tendency of object-based integration on discontinuous sequences, indicating that contextual factors (i.e., how movements are organized and displayed) might have an influence on spatiotemporal integration. Specifically, while spatiotemporal continuity may encourage feature-based representation, spatiotemporal discontinuity may facilitate integration across feature dimensions within individual movement units. Our results showing that the dual-processing cost was significant on continuous (Experiment 1) but not on discontinuous (Experiment 2) sequences when the sequence length was short (i.e., two units) provide evidence for a modulation from contextual factors (i.e., spatiotemporal continuity) on the strength of spatiotemporal integration. From this perspective, the finding of Wood (2007) may be seen as a special case, in which spatiotemporal integration was strengthened by the discrete/discontinuous nature of movement display. The spatial and temporal information, however, are not *automatically* integrated, as demonstrated in Experiment 1.

One may suspect that the lack of dual-processing costs on short discontinuous sequences might result from independent storage capacities for spatial and temporal information, respectively. However, as the spatiotemporal discontinuity should have increased the possibility of object-based rather than feature-based representation (e.g., Wood, 2008), it is not very likely to observe a stronger tendency of parallel processing on discontinuous sequences than on continuous sequences, on which spatial and temporal information had been demonstrated to share a common storage capacity (Experiment 1). Another explanation for the lack of dual-processing costs is that the storage capacity might be sufficient for two units of each information (i.e., four units in total if without integration). Although this might be able to explain the finding in the spatial domain given the close-to-ceiling performance (i.e., low cognitive demand on spatial processing or high storage capacity for spatial information), it cannot explain the data in the temporal domain since the temporal performance was far from the optimal. In other words, any reallocation of cognitive resources from the original temporal processing (i.e., in the *Temporal-only* condition) to the “additional” spatial processing (i.e., in the *Both* condition) should have impaired temporal performance as no spare capacity was available. The finding therefore suggests that a certain level of spatiotemporal integration had occurred during temporal processing.

Nevertheless, the dual-processing cost on four-unit sequences was again significant in both spatial and temporal domains. This might be explained by the fact that encoding movement units defined by a conjunction of features requires a larger capacity (Alvarez & Cavanagh, 2004), and thus retaining four integrated units (compared with two) might have exceeded the cognitive capacity, making the “binding” to fall apart. Alternatively, since the integrated representations are susceptible to interference from subsequent visual stimulation (Allen et al., 2006; Wheeler & Treisman, 2002), the retrospective interference might be higher when more movement units are appended to a sequence, leading to an impairment in the binding structure.

## General discussion

In two experiments, we examined whether spatial and temporal information of human movements are retained as integrated representations or as individual features in working memory and whether spatiotemporal continuity of movement sequences might modulate the process. We found that spatial and temporal information are not automatically and compulsorily integrated. Instead, they are processed separately, but share the storage capacity and compete for a common pool of cognitive resources. Our finding is consistent with the consensus view of the partial independence of memory capacities for different feature dimensions (Schneegans & Bays, 2019), which rejects the strong form of object-based representation in working memory. In addition, we found a tendency of object-based integration on discontinuous sequences, indicating that contextual factors (i.e., how movements are organized and displayed) can further modulate the level of spatiotemporal integration and that presenting movements as isolated units encourages object-based representation.

As discussed previously (see the Discussion section of Experiment 1), perceiving human movements entails an integration of spatial information over time (Giese & Poggio, 2003; Lange et al., 2006), suggesting an intrinsic spatiotemporal dependence in the early stage of movement perception. Nevertheless, the level of spatiotemporal dependence may vary across different stages of information processing or, more specifically, how spatial and temporal information are defined. For example, when temporal information is defined as the change of spatial information over time, such as speed (i.e., the distance travelled along a trajectory divided by elapsed time) or rhythm (i.e., the structure of temporal durations conveyed through a sequence of movements), the processing of temporal information (speed/rhythm) may rely, to some extent, on spatial information (path/trajectory) as being second-order features, while the processing of spatial information can be relatively independent. Consistent with this prediction, Chiou (2022a) demonstrated that temporal processing of human movements required (or co-occurred with) a certain level of spatial processing, but the level of spatial processing can be independently modulated by the focus of attention.

The current results also showed an asymmetric spatiotemporal dependence—namely that the dependence of temporal processing on spatial information was stronger than the dependence of spatial processing on temporal information. Specifically, we found that temporal salience did not modulate spatial encoding on either continuous or discontinuous sequences, indicating that spatial processing is relatively independent from temporal information. But we found a certain level of spatiotemporal integration on discontinuous sequences, especially during temporal processing, illustrating a dependence of temporal processing on spatial information (i.e., a tendency of spatiotemporal co-processing). In fact, the asymmetric spatiotemporal dependence has been widely demonstrated in previous research with more basic stimuli (e.g., lines, dots; Casasanto & Boroditsky, 2008; Casasanto et al., 2010; Dormal & Pesenti, 2013; Santiago et al., 2011; Starr & Brannon, 2016). For example, it has been shown that temporal processing is easily interfered by irrelevant spatial information, but not vice versa (Casasanto & Boroditsky, 2008). This line of research suggests that temporal representation may be intrinsically dependent on spatial representation (Casasanto et al., 2010; Casasanto & Boroditsky, 2008) or that spatial information may be processed more automatically and independently than temporal information due to its higher salience in the visual modality (Dormal & Pesenti, 2013; Santiago et al., 2011; Starr & Brannon, 2016).

The finding of the asymmetric dependence between spatial and temporal processing provides direct evidence against the strong form of object-based representation of human movements. For being an integrated representation, the dependence of temporal processing on spatial information should be comparable with the dependence of spatial processing on temporal information. Moreover, as we can see in the current study, even for temporal information whose processing has been shown to rely on the corresponding spatial information (e.g., Chiou, 2022a), it was not necessarily forming an integrated representation with the spatial information, as illustrated by the significant dual-processing cost in the temporal domain.

One should note that the presence of the dual-processing cost can only reject the strong form of spatiotemporal integration (i.e., integration without additional costs in terms of storage capacity or processing resources) but not all forms of possible integration. For example, integrating features stored in separate domain-specific caches may require additional cognitive resources (Wheeler & Treisman, 2002), leading to a performance decline in the dual-processing condition even when an integrated representation was formed. Moreover, encoding movement units defined by a conjunction of features may require a larger capacity due to higher information load or complexity per unit (Alvarez & Cavanagh, 2004), increasing the cost of processing more features, albeit integrated. However, feature integration was not required by the current task, since the change only occurred in *either* spatial or temporal domain under the dual-processing condition. The partial report method also ensured that only one feature dimension was relevant to memory retrieval, controlling for the decision noise that may have occurred when comparing multiple features. It is therefore reasonable to assume that an integrated representation would only be formed if it is beneficial for the task performance (e.g., by optimizing the encoding process) or is required by the information processing (e.g., due to the intrinsic spatiotemporal dependence in movement perception).

The current research examined whether working memory for human movements are retained as integrated representations or as individual features. This question is crucial especially under the assumption that working memory consists of a fixed number of discrete “slots” in which items are temporarily stored (Cowan, 2001; Luck & Vogel, 1997; Miller, 1956; Zhang & Luck, 2008). For how different types of information are represented in working memory—whether multiple features can be processed as a single unit—strongly determines how much information can be retained. However, the current study did not aim to provide evidence either for or against the slot model of working memory. The finding that spatial and temporal information of movements are not fully integrated but compete for a common pool of cognitive resources can also be explained, for example, by the limited-resource model of working memory (Bays & Husain, 2008; Ma et al., 2014), according to which working memory is conceptualized as a limited resource that is distributed among all items or information to be retained. The limited-resource model would predict a performance decline whenever the cognitive load exceeds the limited capacity of cognitive resources without presuming a fixed number of slots available in working memory.

To conclude, the current study rejects the strong form of object-based representation of human movements in working memory, but supports partial independence of spatial and temporal processing. It also shows that contextual factors, such as the way movements are organized and displayed, can further modulate the level of object-based representation and spatiotemporal integration.

## Data Availability

The datasets generated and analyzed during the current study are available in the PUB repository of Bielefeld University (10.4119/unibi/2966722).

## Footnotes

1 The expertise index (0–4) is defined as the following: 0: No experience, 1: Beginner (skill level = 1 or 2 on a 5-point scale, or skill level > 2 but training length < 3–5 years), 2: Intermediate amateur (skill level = 3 and training length ≥ 3-5 years, or skill level > 3 but training length < 6 years), 3: Advanced amateur (skill level = 4 and training length ≥ 6 years), 4: Professionals (skill level = 5 and training length ≥ 6 years).

2 There were a few exceptions, such as a jump or a circular movement, that were not performed with a bell-shaped velocity profile. Those movements were included into the stimulus set only if a clear starting point and ending point can be identified and the manipulation of movement speed can be precisely implemented.
